# “Grooving in My Body”: A Mixed-Methods Pilot Study of Vibroacoustic Therapy’s Effects on Emotion Regulation and Attention in Autistic Children

**DOI:** 10.3390/healthcare13050465

**Published:** 2025-02-21

**Authors:** Janelle Moore, Kate Farquharson, Carol Lotter

**Affiliations:** Music Therapy Department, School of The Arts, University of Pretoria, Pretoria 0002, South Africa; katefarquharson@gmail.com (K.F.); carol.lotter@up.ac.za (C.L.)

**Keywords:** vibroacoustic therapy, autism, emotion regulation, attention, mixed methods, pilot study

## Abstract

**Background**: Autistic children often face challenges with attention and emotion regulation, which can impact their socio-communication skills and overall well-being. Vibroacoustic therapy (VAT), a sensory-based intervention using low-frequency sound vibrations, may offer a novel approach to address these challenges. The objective of this pilot study is to explore the feasibility and potential efficacy of VAT in improving attention and emotion regulation in autistic children aged 9–12 years. **Methods**: Eighteen children were recruited with assistance from the school psychologist, using purposive sampling to identify participants with autism spectrum disorder and attentional challenges. The study was conducted at a primary school in Pretoria, South Africa, in March 2023. Participants were divided into treatment (n = 9) and control (n = 9) groups. The treatment group underwent 10 VAT sessions over six weeks. Attention was assessed using the NEPSY-II and Joint-Attention Test (JTAT), focusing on sustained, selective, alternating, and joint attention. Qualitative data were collected through observations and creative semi-structured interviews to understand the children’s experiences of VAT. Tests were conducted at baseline, midway, post-intervention, and one week post-intervention. Statistical analysis was conducted using a Generalised Linear Mixed Model (GLMM) in R version 4.3.1, with significance assessed using a Likelihood Ratio Test (*p* < 0.05) to assess attention improvements. **Results**: Quantitative analysis revealed significant improvements in joint attention in the treatment group (X^2^ = 11.64, df = 3, *p* = 0.008). Qualitative findings highlighted positive experiences related to emotion regulation, with children reporting a sense of calm and enjoyment during VAT sessions. Teachers also noted improvements in attention and engagement. **Conclusions**: VAT appears to be a feasible and acceptable intervention for autistic children, with potential benefits for attention and emotion regulation. These findings support further research to validate its efficacy and explore its adaptability for diverse sensory profiles. VAT may hold promise as a holistic therapeutic tool in autism intervention programmes.

## 1. Introduction

### 1.1. Attention and Autism Spectrum Disorder

Autism spectrum disorder is characterised by various symptoms, including difficulties in social communication, repetitive behaviours, and sensory sensitivities [1,2]. Attention difficulties are often overlooked yet significantly impact socio-communication skills [3]. Attention is essential for prioritising relevant thoughts while filtering distractions [4]. Autistic children often exhibit structural brain differences that hinder attention [3]. Sohlberg and Mateer (2001) identify five types of attention: focused, sustained, selective, alternating, and divided. In addition to these, autistic children often face challenges with joint attention—a related and critical aspect of social interaction that involves sharing focus with another person on an object or event [5,6] (Table 1). For the purposes of this study, we focus on specific aspects of attention, including sustained, selective, alternating, and joint attention. These attentional challenges are compounded by other autism characteristics, such as sensory sensitivities, restricted interests, and difficulties with social communication, which further hinder the development of socio-communication skills [1,2].

### 1.2. The Link Between Attention and Emotion Regulation

Another common challenge for autistic children is emotion regulation—the ability to manage and control moods and feelings—which is important for overall well-being [8,9,10,11]. Difficulties with emotion regulation can intensify challenges in social communication and interaction, further affecting well-being [12].

Research highlights a reciprocal relationship between emotion regulation and attention, with effective emotion regulation being essential for optimising attentional capacity [12,13,14,15]. Conversely, emotion dysregulation can hinder attention [16]. Continuous exposure to safe environments can enhance emotion regulation skills, improving attention to thoughts and feelings and supporting overall emotional well-being [12]. Studies also suggest that attentional difficulties may contribute to emotion-regulation challenges in autistic individuals [1,17,18]. Enhancing attention may foster neuroplasticity, leading to better emotion regulation and overall well-being [14,15,19,20].

### 1.3. Music Therapy and Autism

Research indicates that certain types of music, when carefully selected or composed, can help promote calm and support emotion regulation in autistic children [13]. Calming music—characterised by gentle melodies and predictable rhythms—has been shown to reduce overstimulation and facilitate relaxation [16,21,22]. Additionally, music that resonates with aspects of a dysregulated state may serve as a precursor to guiding individuals toward a calmer state by meeting them where they are emotionally [23].

Music therapy, a healthcare profession that uses music within therapeutic relationships to address physical, emotional, cognitive, and social needs, extends these benefits through individualised and evidence-based interventions. Techniques such as Neurologic Music Therapy (NMT), including Music Attention Control Training (MACT), actively engage brain networks linked to emotional well-being and attentional control, showing promising outcomes for autistic children [3,24]. Unlike passive music listening, these interventions involve structured, goal-oriented engagement facilitated by a qualified music therapist. However, a gap remains in research focused on receptive music-therapy techniques—where the client primarily listens to music rather than actively participating—specifically targeting emotion regulation and attention deficits within this population.

### 1.4. Vibroacoustic Therapy (VAT)

For centuries, various cultures worldwide have utilised sound vibrations for healing across physical, mental, and spiritual dimensions [25]. Vibroacoustic therapy (VAT) is a non-invasive and receptive form of music therapy that uses low-frequency sinusoidal vibrations delivered through specialised equipment, including chairs, beds, pillows, and a “sound mat” that users lie on [26]. This therapy is administered by trained health professionals and has shown effectiveness in treating conditions such as fibromyalgia, Parkinson’s disease, insomnia, and depression [27,28,29]. VAT was co-pioneered in the 1980s by Olav Skille in Norway and Petri Lehikoinen in Finland, building on earlier practices of vibration therapy. These included 18th- and 19th-century innovations, such as vibrating chairs and tuning forks, used for health conditions like melancholia and migraines [30,31]. Skille focused on the effects of specific low frequencies on relaxation and muscle tone, while Lehikoinen developed systems like the NextWave chair to enhance circulation and muscle resonance [32,33]. Sessions typically last 15 to 40 min, using frequencies between 25 and 75 Hz, ensuring relaxation without overstimulation [34].

Vibroacoustic therapy (VAT) operates through sympathetic resonance, where sound vibrations elicit responses in the body that influence muscle tone, blood pressure, and heart rate, promoting relaxation [22]. By transducing sound vibrations into mechanical stimulation, VAT impacts tissues and mechanoreceptors similarly to other vibratory inputs [35]. The integration of specific-frequency music during VAT sessions enhances relaxation, with evidence suggesting that particular sound frequencies can modulate neural responses critical for neuroplasticity and cognitive functions [22,31]. Notably, 40 Hz gamma oscillations have been associated with neural processes supporting attention and neuroplasticity [35]. Studies indicate that VAT, incorporating low-frequency vibrations like 40 Hz, may help regulate irregular brain oscillations linked to attention deficits in autistic individuals [27,36]. Nevertheless, further research is needed to elucidate the mechanisms and clinical relevance of 40 Hz stimulation in VAT for improving attention and emotion regulation in this population.

### 1.5. VAT and Autism

Research on VAT’s effects on attention in autistic children is limited but promising. Skille (1989) reported improved tolerance of physical contact in children with tactile defensiveness after VAT [33]. Similarly, Shirazi et al. (2023) demonstrated improvements in the emotional profiles of autistic children when VAT was incorporated into a sensory rehabilitation program [37]. In autistic adults, VAT has been associated with increased relaxation and decreased aggression [38]. However, more research is needed to understand its specific effects on children’s attention.

One of VAT’s strengths lies in its ability to attune to an individual’s current emotional or physiological state through low-frequency sound vibrations. By “meeting” individuals in their dysregulated state, VAT provides a non-invasive approach to engage their sensory and emotional systems. This initial attunement fosters a sense of safety and validation, which can serve as a precursor to guiding them toward a more regulated and calmer state.

Qualitative research highlights VAT’s emotion-regulating effects, suggesting improvements in concentration and emotional stability [37,39]. It may also promote mindfulness, enhancing present-moment awareness and relaxation [40]. Additionally, the neurophysiological basis of VAT supports these mechanisms, as it may regulate brain oscillations linked to attention, potentially enhancing joint attention [27,41].

### 1.6. Current Study

While VAT demonstrates potential in addressing attention deficits and enhancing emotion regulation, there is a significant gap in research focused specifically on its impact on autistic children. This paper aims to explore the potential of VAT as an intervention to address both emotion regulation and attention difficulties in autistic children. The following research questions guided this study:

1. Quantitative question: Can VAT improve attention in autistic children?
2. Qualitative question: How do autistic children experience VAT?
3. Mixed-methods question: Is VAT an effective and practically feasible intervention for autistic children?

## 2. Materials and Methods

### 2.1. Study Design

**Figure 1:**
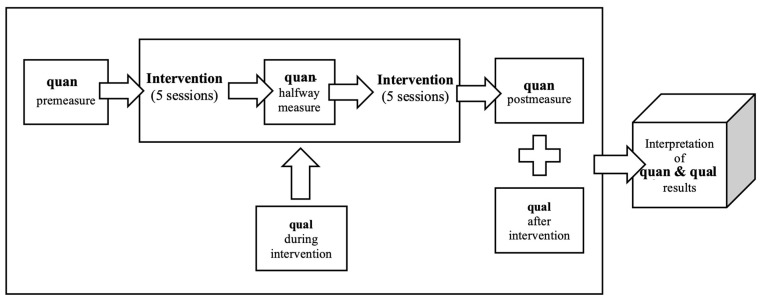
Concurrent design.

This study employed a concurrent mixed-methods design, involving three groups: a treatment group receiving VAT, a control group with no treatment, and a pilot group which assisted in refining the procedure during the pilot phase before the main intervention began. Quantitative and qualitative data were collected separately and concurrently over a six-week period and later integrated during the discussion phase (Figure 1). Rooted in a pragmatic paradigm, this approach emphasised practical insights, valuing both participants’ perspectives in the qualitative analysis and quantitative outcomes to assess the intervention’s effectiveness [42,43]. The first author collected all data and facilitated the VAT intervention.

### 2.2. Participants

This study was conducted at a quintile 3 primary school in Pretoria, South Africa, which serves children from low-socioeconomic backgrounds and does not charge fees [44]. The study commenced in March 2023. Eighteen children, aged 9–12 years, diagnosed with autism spectrum disorder (ASD) and exhibiting attentional challenges (sustained, selective, or alternating attention difficulties), were recruited with the assistance of the school psychologist. To ensure comparability between the treatment and control groups, participants were matched by the school psychologist based on key characteristics, such as verbal ability, cognitive functioning, and level of attentional challenges. The matching process was based on the psychologist’s professional knowledge of the children’s abilities and challenges, ensuring that both groups were as similar as possible with respect to these factors. This process resulted in two groups of 9 participants each: the treatment group (n = 9) and the control group (n = 9). The control group received no treatment or placebo during the study period; it served as a baseline for comparison with the treatment group and helped mitigate the practice effect. Ethical approval from the University of Pretoria, Faculty of Humanities Ethics Committee and guardian consent were obtained, and each child provided verbal and written assent after a comprehensive explanation of the study, which included pictures and visual demonstrations to aid understanding.

Inclusion criteria required participants to have sufficient verbal communication skills to ask questions, respond to brief feedback, and indicate if they wished to pause or stop the sessions. Additionally, the school psychologist ensured no participants had contraindicated conditions for VAT, such as pacemakers or acute physical conditions [28].

### 2.3. Pilot Phase

In preparation for the main study, the first author conducted pilot sessions with three participants, who were not part of the final study but only involved in the pilot phase, to assess their engagement and ability to sustain focus for 20 min during the VAT intervention. This session took place in the same setting as the treatment group, one month before the main study began. The children were able to comfortably lie on the VAT mat for the duration. To help one participant with time awareness and reduce restlessness, a car-racing video, as a visual cue, was used, which proved effective. Although additional stimulation tools were available, they were not needed. Consultation with Dr. Lee Bartel confirmed these tools would not affect data integrity. The NEPSY-II and JTAT assessments were administered for the purpose of the first author familiarising herself with their use, while under the supervision of the school psychologist.

### 2.4. Procedure

The researcher communicated the study details to the children using clear, age-appropriate language, emphasising the voluntary nature of participation and the option to withdraw at any time. Participants provided both verbal and written assent.

The treatment group underwent ten 20-min VAT sessions using the Sound Oasis VTS1000 equipment (Marblehead, MA, USA), facilitated by a music-therapy student [45]. During each session, participants listened to a 40 Hz frequency song through headphones while experiencing 40 Hz sound vibrations through a mat. Sessions were conducted individually, twice weekly for five weeks. Throughout, participants were monitored for signs of discomfort, and a stop sign was provided for participants to indicate if they wished to stop. Occasionally, signs of restlessness were observed, which were addressed by introducing additional activities to maintain engagement without introducing confounding variables.

Although no risks were identified, the researcher remained mindful of the potential for distress due to the unfamiliarity of VAT. The school psychologist was available for emotional support, though no participants required assistance, as there were no adverse effects from the intervention. Sound levels were carefully monitored to avoid discomfort, with the volume never exceeding 70 dB(A).

Confidentiality was maintained by assigning numerical labels to participants, and all data were stored securely at the University of Pretoria and will remain there for at least 10 years. Participants in the control and pilot groups were informed that they could request up to 10 VAT sessions after the study, though no participants made such requests.

Attention assessments (NEPSY-II and JTAT) were administered at four time points: pre-test (baseline), mid-test (after five sessions), post-test 1 (immediately after 10 sessions), and post-test 2 (one week after the final session). Teacher questionnaires were completed before and after the intervention. Qualitative data were collected throughout the intervention period, with creative interviews conducted following the final session. A diagram of the procedure is provided in Appendix A.

### 2.5. Measures

#### 2.5.1. Quantitative Measures

- NEPSY-II: Second Edition of the Developmental Neuropsychological Assessment—Auditory Attention (AA) and Response Set (RS): The NEPSY-II is a standardised neuropsychological assessment designed to evaluate cognitive functioning in children aged 3 to 16 years [46]. This study utilised the Auditory Attention (AA) and Response Set (RS) subtests to assess sustained, selective, and alternating attention. Both subtests have demonstrated high validity and reliability in evaluating attentional capacities in children [47,48].
- Joint-Attention Test (JTAT): The JTAT is a standardised measure designed to assess joint attention in children, particularly those with ASD, through interactive tasks that mimic natural social interactions [49]. It is easy to administer and provides reliable insights into joint-attention behaviours. Adaptations to the original procedure were made for this study to align with its context while maintaining validity.
- Teacher questionnaires (7-item Likert scale): Teachers completed a questionnaire before and after the intervention, which included seven Likert-scale questions based on established definitions of attention and joint attention [5,7]. These items were designed to capture observed changes in attentional behaviours with high consistency.

#### 2.5.2. Qualitative Measures

- Observation notes during VAT sessions: Detailed notes were taken during each session to document participants’ physical and verbal responses to the intervention.
- Brief feedback after odd-numbered sessions: Participants provided feedback after sessions 1, 3, 5, 7, and 9, offering insights into their experiences with VAT.
- Creative semi-structured interviews post-intervention: After the final session, participants engaged in creative interviews involving drawing, music-making, and other expressive activities. This approach allowed participants to convey their experiences through diverse mediums, enhancing accessibility and depth of qualitative data [50].

### 2.6. Data Analysis

The data analysis involved both quantitative and qualitative approaches. Quantitative data, including NEPSY-II and JTAT scores, as well as Likert-scale responses from teacher questionnaires, were analysed using a Generalised Linear Mixed Model (GLMM), with the assistance of a statistician. Participant 10’s data were excluded due to withdrawal, and Participant 2’s data were analysed both with and without their missed sessions. Since both analyses yielded the same results, the second analysis was discarded.

Qualitative data were analysed through thematic analysis, where video recordings from semi-structured interviews and observation notes were transcribed, coded, and categorised to identify patterns and themes. Despite excluding Participant 10’s quantitative data, their qualitative data from the first two sessions were included in the analysis.

The integration of both data sets was crucial for a comprehensive understanding of VAT’s effectiveness in improving attention in autistic children. The findings were examined for convergence or divergence during the discussion chapter, providing a holistic view of the intervention’s impact. Feasibility was assessed qualitatively through observations of participant engagement, relaxation, and responsiveness to VAT. These observations also guided considerations for potential adaptations to enhance comfort and engagement, ensuring VAT could be tailored to individual sensory preferences.

### 2.7. Research Quality

Ensuring research quality in mixed-methods research remains debated [51], but triangulation of multiple data sources is widely recognised as contributing to reliability [52]. This study integrated qualitative and quantitative data to provide a comprehensive understanding of VAT’s feasibility for autistic children. Triangulation was applied through various attention tests, including the NEPSY-II and JTAT assessments, both known for their high validity and reliability [47,53]. A control group helped mitigate the practice effect [54].

Validity, which refers to the accuracy of measurement, was ensured throughout the study [55]. A pilot session with three participants familiarised the first author with the equipment, refined procedures, and enhanced the study’s validity [56].

Although the small sample size limits generalisability and statistical power, it allowed for in-depth investigation and qualitative insights. A pilot study’s role is to inform larger-scale research rather than generalise findings. The complex nature of ASD necessitates a person-specific approach to VAT, as evidenced by adaptations made to engage participants who showed restlessness. These modifications, such as introducing playdough or timer videos, were not seen as confounding variables but as necessary adjustments to maximise engagement [39]. While some uncontrollable factors, like the time of day of assessments, may have influenced results, these issues are further explored in the Conclusion.

## 3. Results

### 3.1. Quantitative Data Analysis

#### 3.1.1. NEPSY-II 2nd Edition—Auditory Attention (AA)

The NEPSY-II Auditory Attention (AA) subtest assessed sustained and selective attention, with scores ranging from 1 to 15. No significant interaction between treatment and time was found (X^2^ = 0.69, df = 3, *p* = 0.875), nor was there a significant treatment effect (X^2^ = 0.025, df = 1, *p* = 0.874) (Figure S1). On average, the control group scored 0.69 units lower than the treatment group across all time points (control: 5.22 ± 1.41; treatment: 5.92 ± 1.41). The mean score differences ranged from 0.33 (pre-test) to 0.89 (mid-test and post-test 1).

A significant difference in Auditory Attention scores over time was found (X^2^ = 28.16, df = 3, *p* < 0.001), suggesting a practice effect. No treatment effect was observed, but time-based comparisons showed no significant differences between pre-test and mid-test scores (control: β = −0.44, t = −0.98, *p* = 0.323; treatment: β = −1.00, t = −1.62, *p* = 0.109). Significant increases were found between pre-test and post-test 1 (control: +1.44, t = −2.84, *p* = 0.006; treatment: +2.00, t = −2.85, *p* = 0.005), and between pre-test and post-test 2 (control: +1.67, t = −3.58, *p* < 0.001; treatment: +2.00, t = −3.17, *p* < 0.001). Despite these changes, no immediate, dose, or sustained treatment effects were found, with both groups showing similar trends.

#### 3.1.2. NEPSY-II 2nd Edition—Response Set (RS)

The NEPSY-II Response Set (RS) subtest measured sustained and alternating attention, with scores ranging from 1 to 15. No significant interaction was found between treatment and time (X^2^ = 0.384, df = 3, *p* = 0.943), and no treatment effect was observed (X^2^ = 0.003, df = 1, *p* = 0.951) (Figure S2). On average, the control group scored 0.16 units lower than the treatment group (control: 3.92 ± 1.14; treatment: 4.08 ± 1.16). The mean differences between groups ranged from −0.33 (post-test 1) to 0.11 (pre-test).

There was a significant difference in response subset scores over time (X^2^ = 17.26, df = 3, *p* < 0.001), suggesting a practice effect. Time-based comparisons showed no significant differences between pre-test and mid-test scores (control: β = −0.44, t = −1.49, *p* = 0.193; treatment: β = −0.78, t = −1.44, *p* = 0.154). Significant increases were found between pre-test and post-test 1 (control: +1.22, t = −2.81, *p* = 0.007; treatment: +1.67, t = −2.12, *p* = 0.039) and pre-test and post-test 2 (control: +1.56, t = −3.36, *p* = 0.001; treatment: +1.89, t = −2.25, *p* = 0.028). Despite these changes, no immediate, dose, or sustained treatment effects were found, with both groups showing similar trends.

#### 3.1.3. JTAT Scores

The JTAT evaluation assessed joint-attention changes over time, with four phases: comparing pre-test and mid-test scores for immediate and dose effects, post-test 1 for treatment and immediate effects, and post-test 2 for sustained effects. JTAT scores ranged from 1 to 25.

**Figure 2:**
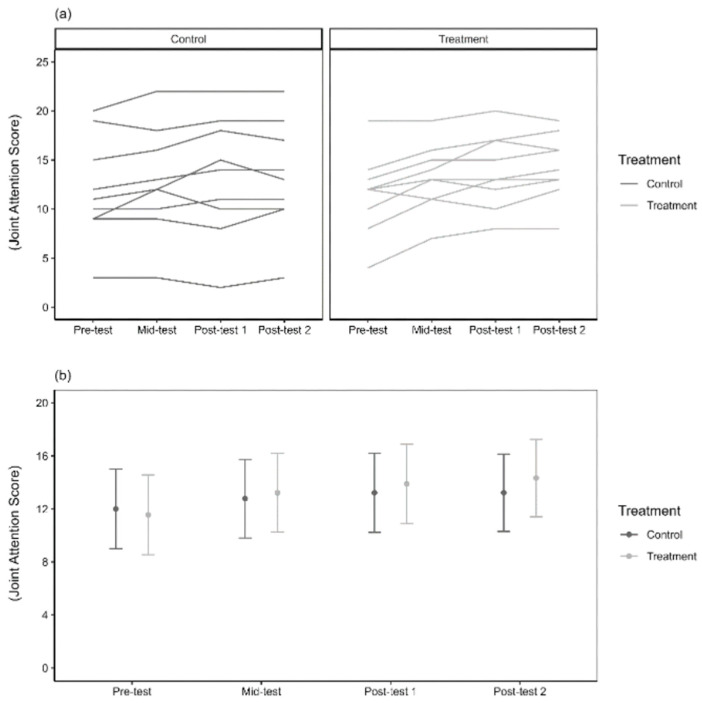
JTAT joint-attention scores. Panel (**a**) represents the raw scores for nine individual subjects per treatment and control groups at four different time intervals, while panel (**b**) indicates the marginal predicted means (±95% confidence interval of the mean) across the four different time intervals.

A significant interaction was found between treatment and time (X^2^ = 11.64, df = 3, *p* = 0.008), indicating that joint-attention scores changed differently over time between the treatment and control groups (Figure 2). This was primarily due to a 1.67-unit higher score in the treatment group by mid-test (t = −3.57, *p* < 0.001), while the control group showed no significant change (β = −0.78, t = −1.74, *p* = 0.086). Joint-attention scores were significantly higher in both groups at post-test 1, with the treatment group showing a 1.21-unit greater change compared to the control group. At post-test 2, the treatment group had a 1.56-unit greater improvement than the control group.

These results suggest a dose effect after five sessions, a treatment effect after 10 sessions, and sustained effects one week later. Immediate effects remain uncertain, as both mid-test and post-test 1 reflect cumulative effects from multiple sessions. To assess immediate effects more accurately, assessments immediately after the first session would be needed. The timing of VAT’s impact on joint attention is unclear, as improvements were seen after five sessions, and earlier assessments were not conducted. Future studies could benefit from more frequent assessments, and comparing mid-test to post-test 1 could help determine if five sessions were sufficient to optimise joint attention.

#### 3.1.4. Questionnaire Data

**Figure 3:**
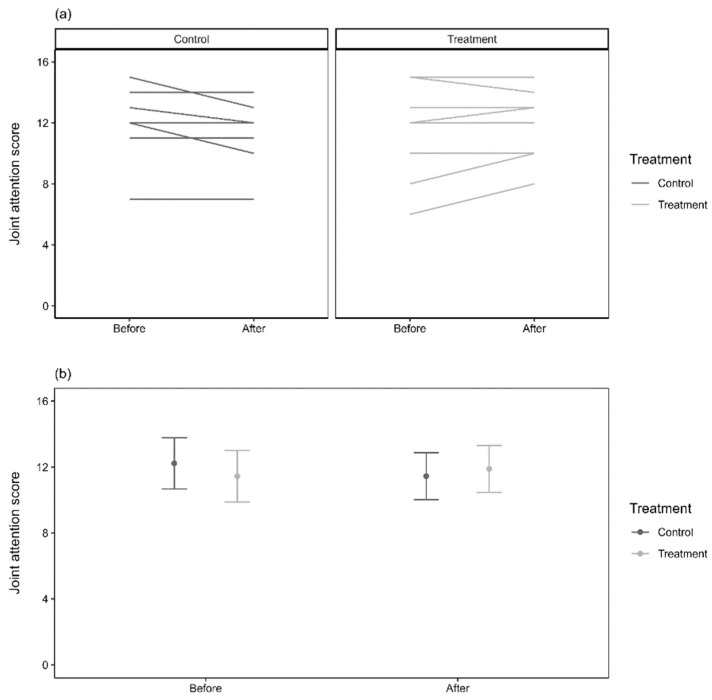
Joint-attention scores. Panel (**a**) represents the raw scores for nine individual subjects per treatment and control groups before and after treatment interventions, while panel (**b**) indicates the marginal predicted means (±95% confidence interval of the mean) before and after treatment interventions.

Teacher questionnaires indicated no effect on overall, sustained, selective, or alternating attention (Figures S3–S6). However, there was a significant interaction between treatment and time for joint attention (X^2^ = 0.28, df = 1, *p* = 0.598) (Figure 3).

### 3.2. Qualitative Results

**Figure 4:**
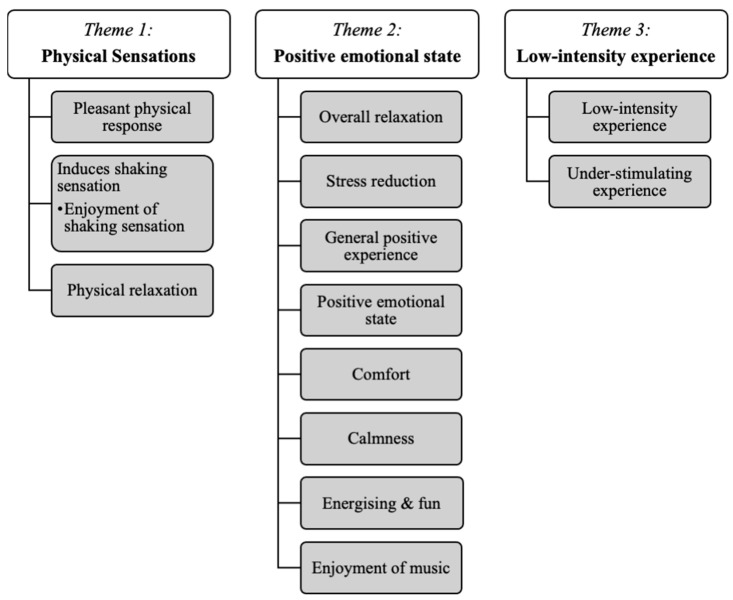
Themes and categories.

Thematic analysis identified three main themes: physical sensations, positive emotional state, and low-intensity experience, which collectively highlighted children’s engagement and responses to VAT (see Figure 4).

#### 3.2.1. Theme One: Physical Sensations

Children often described various physical sensations experienced during VAT. Common descriptions included a shaking sensation and a sense of relaxation. For example, Participant 2 mentioned enjoying how it “shook” him and likened it to a massage. Participant 3 expressed that it felt like it was “grooving in my body”. Many children also associated warmth, comfort, and relaxation with the experience. In the creative interviews, participants used drumming, movement, and vocalisations to convey these physical sensations, often linking them with positive emotions.

#### 3.2.2. Theme Two: Positive Emotional State

**Figure 5:**
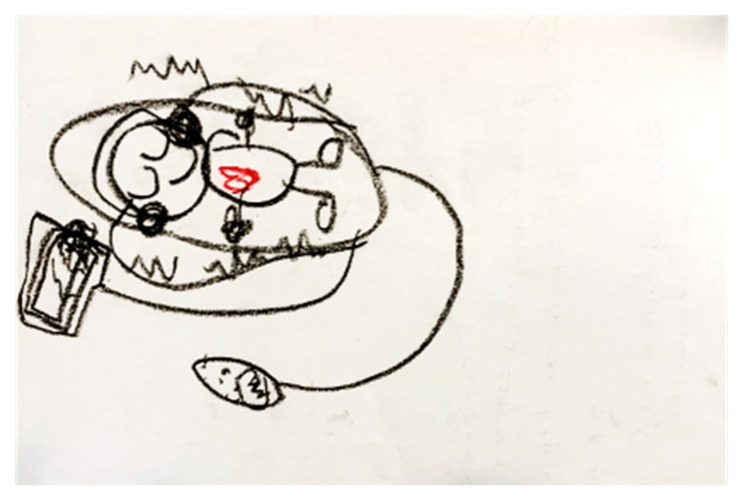
Participant 2’s drawing showing enjoyment.

Children frequently expressed positive emotions during VAT sessions, using words like “nice”, “good”, “comfortable”, “calm”, and “relaxed”. Some felt energised and joyful, showing enjoyment through movement, songwriting, and music-making. For example, Participant 2 adapted “Jingle Bells” lyrics to reflect their enjoyment and created a drawing that conveyed similar feelings (see Figure 5). Participant 7 self-hugged to express warmth, and many participants laughed and smiled, further indicating enjoyment and relaxation. Notably, Participant 1, who experienced a pre-session meltdown, reported feeling “better” afterward.

#### 3.2.3. Theme Three: Low-Intensity Experience

This theme captures categories reflecting low-intensity and gentle experiences. Participant 3 described VAT as “soft”, comparing it to a snail’s slow and non-aggressive qualities, illustrated by a snail sculpture they created during the creative interview. Some children also used gentle movement, singing, and instruments to convey VAT as a low-intensity experience. However, some participants found it understimulating; for example, Participant 2 reported feelings of boredom, and both Participants 2 and 7 exhibited restlessness, frequently asking about the time. When provided with additional activities, such as clay and a timer video during the VAT session, they were no longer restless.

## 4. Discussion

### 4.1. Significant Effects on Attention

The observed improvements in joint attention across testing phases align with previous research highlighting similar effects following music-therapy interventions, which primarily focused on active music-making rather than VAT’s receptive approach [57,58,59]. These improvements may partly stem from VAT’s emotion-regulating effects, given the well-established connection between emotion regulation and social engagement. Since social engagement is closely tied to joint attention [12,41,60], VAT’s calming and regulatory influence may indirectly support joint-attention abilities.

On a neurophysiological level, the superior temporal sulcus (STS)—a key region for processing social cues and coordinating joint attention—operates optimally at gamma frequencies around 40 Hz [41,61]. This region integrates visual, auditory, and social information, allowing for rapid detection and response to social stimuli [41,61]. Autistic individuals often exhibit atypical connectivity in the STS and surrounding networks, leading to difficulties in processing social cues and coordinating attention [61]. Recent studies suggest that 40 Hz sound stimulation can help regulate and entrain irregular neural oscillations, potentially improving synchronisation within these networks [27,28,62].

Gamma oscillations are known to play a crucial role in higher-order cognitive processes, such as selective attention, working memory, and perceptual binding [63,64]. Specifically, 40 Hz stimulation may enhance functional connectivity between the STS and prefrontal regions involved in attention control, allowing for more efficient information integration during joint-attention tasks. This neural entrainment effect could explain the observed improvements in joint attention in this study.

However, it is important to recognise that joint attention relies on a broader network involving multiple frequency bands and regions beyond the STS. While gamma oscillations may facilitate perceptual integration, other aspects of joint attention—such as sustained attention and the inhibition of competing stimuli—are associated with lower frequency bands, such as theta and alpha [58,59]. VAT’s dominant 40 Hz stimulation may not fully engage these additional frequency bands, which could limit its overall impact on more complex attentional functions.

This highlights the need for further investigation into the precise mechanisms by which different frequency-specific sound vibrations influence brain networks. Future studies may benefit from employing neuroimaging techniques, such as EEG or fMRI, to explore how VAT-induced neural entrainment affects connectivity in the STS and related attention networks in real time. This will provide a more comprehensive understanding of how VAT influences neural circuits underlying joint attention and social communication.

### 4.2. Children’s Emotion Regulating Experiences of VAT

#### 4.2.1. Relaxation and Stress Reduction

According to Porges (2017), a state of physical and emotional calmness reflects a degree of emotion regulation, which may enhance social engagement and joint attention [12]. Many participants in this study reported feelings of calmness, relaxation, and comfort during VAT, which aligns with previous findings on the relaxing effects of VAT for children with ASD [33,37]. Some participants also noted a reduction in stress during and after sessions, suggesting VAT’s potential to alleviate tension and promote emotional regulation. Instances of participants falling asleep further underscore VAT's capacity for deep relaxation, which may reflect a downregulation of heightened arousal states [65].

Although not all instances were followed by immediate joint-attention assessment, this emotional regulation likely contributed to participants’ improved engagement. This aligns with research that highlights VAT’s role in calming heightened emotional states and supporting social connection [33,39]. These findings suggest that relaxation and stress reduction during VAT may have played a key role in enhancing joint attention.

#### 4.2.2. Sensory Integration

VAT may support sensory integration, a key factor in emotion regulation for autistic children [66,67]. Autistic children often experience challenges with tactile and auditory sensory input [68,69]. The gentle vibrations in VAT can help regulate sensitive tactile systems, promoting physical comfort and a sense of safety [33,39]. Participants’ experiences of warmth and comfort, also displayed by self-hugging, also aligns with Skille’s (1989) findings that VAT can reduce tactile defensiveness and improve receptivity to touch [33].

Similarly, the consistent, soothing music, which many participants reported as being relaxing, in VAT may facilitate auditory integration and minimise overstimulation [18,39,67,70]. Participants’ reports of VAT being calm and relaxing may suggest that the combination of tactile and auditory stimuli plays a role in regulating arousal and enhancing emotion regulation. Improved sensory integration may also contribute to greater joint attention and social engagement [12,71].

#### 4.2.3. Mindfulness

Mindfulness-based interventions support emotion regulation in autistic by enhancing present-moment awareness and bodily focus [71,72]. VAT is considered a mindfulness-based practice, encouraging attention to physical sensations and promoting relaxation [40].

In this study, participants described VAT sensations as “moving all over”, “grooving”, and “like having a massage”, evoking warmth and calm. Responses such as “I cannot feel any stress in my head anymore” and “It feels clear now” reflect increased self-awareness and a connection to the present moment, key aspects of mindfulness [73]. Although the children did not intentionally practice mindfulness, VAT fostered similar outcomes by redirecting their attention to bodily sensations, helping reduce distress and enhance emotion regulation.

#### 4.2.4. Therapeutic Presence

**Figure 6:**
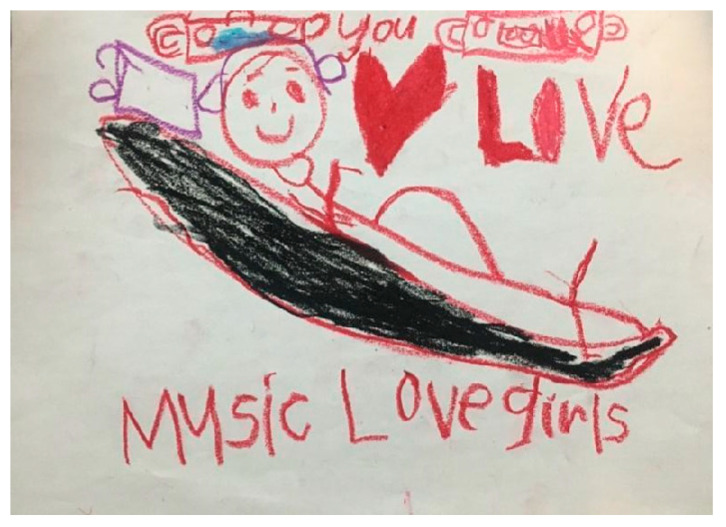
Participant’s drawing.

The consistent therapeutic presence during VAT sessions may have contributed to the observed improvements in joint attention and emotion regulation. A participant’s drawing with the words “girl”, “love”, and “music”, directly referencing author one/the therapist (Figure 6), seemed to suggest a positive connection. Although VAT was the primary intervention, my role included escorting children to sessions, conducting regular check-ins, and maintaining a warm, empathetic demeanour. These interactions may have fostered a sense of safety and trust, creating an environment conducive to emotional regulation and engagement.

Porges (2017) emphasises the importance of therapeutic presence in co-regulation, where nonverbal cues such as facial expressions and vocal tone convey safety and understanding. This sense of relational safety is vital for autistic children, who may struggle with emotional regulation [12]. Punkanen et al. (2017) highlight that even in moments of emotional dysregulation, a strong therapeutic relationship can help children find comfort and stability, amplifying the benefits of VAT [39].

Given the established link between emotion regulation and joint attention [12,60], the therapeutic relationship likely played a complementary role in supporting the children’s engagement. My consistent presence may have provided an anchor, facilitating the children’s ability to remain calm and focused during sessions, ultimately enhancing the intervention’s effectiveness.

#### 4.2.5. Enjoyment and Engagement

Participants consistently demonstrated positive emotional responses, such as joy and excitement, aligning with Ahonen et al.’s (2012) findings on the energising effects of music interventions [40]. Enjoyment may also enhance the efficacy of therapeutic interventions, as playfulness and positive emotional states are more accessible when individuals are emotionally regulated [12]. While some participants required additional activities to maintain engagement, these adaptations highlight the importance of flexible intervention strategies tailored to individual preferences and needs.

### 4.3. Other Notable Findings

Children’s responses to VAT included interesting effects beyond emotion regulation. Experiences of warmth, such as Participant 7’s self-hugging and sun-themed drawings, hint at VAT’s calming physical impact, potentially due to the resonance of sound vibrations with the body, as noted in previous studies [28,39,74,75]. Children’s gentle and non-aggressive portrayals of VAT through movement and artwork further suggest that VAT provided sensory-friendly stimulation, accommodating ASD-related sensitivities.

While relaxation was a common response, some participants showed signs of restlessness, indicating a need for additional engagement. Supplementary activities like playdough and videos effectively engaged three participants, aligning with Punkanen et al.’s (2017) findings on enhancing engagement with VAT [39]. Participant 2’s occasional expressions of boredom also suggest that adding more stimulating elements could enhance involvement.

An improvement in expressive ability was also observed in Participant 1, who entered the session visibly upset by unrelated issues but displayed increased calmness and clearer speech after VAT, expressing his feelings and noting he felt “better”. Although anecdotal, this finding aligns with Ellis’s (2004) research on VAT’s potential to enhance verbal communication in older adults [76]. Given the link between emotion-regulation and speech-production challenges in autistic children, further investigation into VAT’s effects on speech in this population is recommended [3,12].

### 4.4. Non-Significant Findings

The nuanced results in other attention types—sustained, selective, and alternating—highlight attention’s complexity. Although improvements were noted, their lack of statistical significance contrasts with other studies on music-listening interventions [3,77,78]. This discrepancy may reflect frequency-specific neural mechanisms: while VAT’s 40 Hz gamma stimulation appears effective for selective attention, other attention types may engage different frequency bands [35,63,64]. Tailoring frequency selection may enhance future interventions.

The NEPSY-II subtests posed challenges in isolating specific attentional domains, as the Auditory Attention subtest measured both sustained and selective attention, while the Response Set subtest assessed sustained and alternating attention together. These combined measures may have limited the analysis’s precision for each domain. Additionally, the teacher questionnaires provided only one question per attention domain, reducing internal validity for domain-specific comparisons.

Research suggests that while gamma frequency stimulation enhances selective attention, sustained attention relies more on theta frequency, and alternating attention involves both theta and gamma frequencies [79,80]. This frequency difference may partially explain the non-significant findings for sustained and alternating attention.

## 5. Conclusions

### 5.1. Feasibility of VAT Intervention

VAT shows promise for autistic children, particularly in supporting joint attention and emotion regulation, but it may require tailoring to individual sensory needs. While the 40 Hz music used in this study was chosen for its neurological effects, some participants experienced restlessness, suggesting that incorporating client-selected music or a combination of options could improve engagement and enjoyment. Adapting VAT with additional activities, such as playdough or visual timers, could also benefit children requiring more stimulation, creating a more interactive and comfortable setup. Strengthening the therapeutic relationship may further enhance emotional regulation, especially for participants who appeared restless.

Adjusting session length, frequency, or vibration intensity could also increase effectiveness. Although this study was limited to 10 sessions of 20 min each, longer sessions might yield greater benefits. Gradual increases in volume and intensity accommodated sensory sensitivities, but higher levels could potentially benefit some children when tailored to their individual needs. VAT, therefore, appears feasible as part of a broader therapeutic programme for autistic children, with potential for improvement through personalised adjustments and integrated activities that foster holistic engagement. Future research should explore these sensory adaptations and investigate the role of VAT in conjunction with other therapies to achieve more targeted outcomes.

### 5.2. Practical Implications

This study contributes to the limited literature on VAT for autistic children. More research is needed to better understand VAT’s impact on emotion regulation and attention, especially as this was a small pilot study. However, given that VAT shows promise as an implementable intervention, there are several practical implications for its future use:

- Educational settings: Integration into sensory rooms within schools for autistic children.
- Clinical applications: Incorporation into multimodal therapy plans within healthcare settings.
- Home-based implementation: Potential adaptation as a daily practice similar to mindfulness and meditation routines.

### 5.3. Strengths and Limitations

This pilot study offers valuable insights into the effects of vibroacoustic therapy (VAT) on attention in autistic children, despite having some limitations. A key strength lies in its mixed-methods approach, which provided a richer understanding of participant experiences by combining quantitative measures with qualitative feedback obtained through creative activities like drawing and music-making. The small sample size allowed for more in-depth analysis and engagement with the data, enhancing the study’s exploratory value. Additionally, the experimental design incorporated robust elements, including random selection of participants, matching to control for socioeconomic factors, a control group, and multiple testing points, which aimed to strengthen the validity of findings.

However, the small sample size of nine per group remains a significant limitation, reducing the generalisability and statistical power of the results [81]. Uncontrolled variables, such as testing times and medication use, introduced potential biases, with afternoon assessments often coinciding with reduced focus due to tiredness, as noted by teachers. Instrumentation challenges arose from the inclusion of children with mild intellectual disabilities, which may have affected NEPSY-II test results due to ceiling or floor effects. Furthermore, participants from low-income backgrounds may have faced difficulties understanding the tests, though matching efforts were made to mitigate this.

The study also acknowledges that the first author was involved in all data collection and VAT intervention procedures, whilst being a music-therapy student. As a result, there was no blinding in the assessment process. While this provided valuable clinical expertise and insight, the researcher’s direct involvement may have introduced bias. Future studies should consider using blinded assessors to minimise potential bias.

Furthermore, this study’s setting in a single school serving children from low socioeconomic backgrounds may affect the generalisability of the findings. Future research should include participants from diverse settings to improve applicability.

### 5.4. Recommendations for Future Research

This study offers several recommendations for future research to deepen understanding of VAT for autistic children:

- Larger sample sizes: Future studies should include more participants to improve statistical power and generalisability beyond this pilot study.
- Quantitative focus on emotion regulation: Beyond attention, future research could assess VAT’s impact on emotion regulation using specific emotion-regulation measures.
- Immediate and cumulative effects: To distinguish immediate from cumulative effects, initial sessions should be assessed separately, and joint attention could benefit from more frequent testing to clarify VAT’s impact after various session intervals.
- Extended and long-term studies: Lengthening intervention periods and monitoring participants over time would help determine the sustainability of VAT’s effects on joint attention and emotional states.
- Tailored VAT interventions: Exploring adaptations based on sensory profiles—such as music choice, vibration intensity, and session length—may help refine VAT for meeting individual needs.
- Sleep and therapeutic integration: Given its potential effect on sleep, VAT’s impact on sleep patterns could be a valuable focus. Future research could also examine how VAT complements other therapies, enhancing holistic approaches for autistic children.
- Creative data collection: Building on this study’s use of creative interviews, future research could refine these methods to better capture the experiences of children with communication challenges.

## Figures and Tables

**Table 1:** Sohlberg and Mateer’s Attentional Model [7].

Attention Domain	Description
Focused attention	Response to distinct stimuli
Sustained attention	Maintenance of focus over time
Selective attention	Focus in the presence of distractions
Alternating attention	Ability to switch focus between tasks
Divided attention	Attention to multiple stimuli/tasks

**Figure A1:**
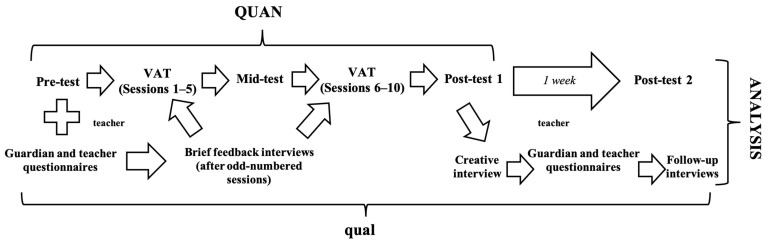
Procedure for treatment group.

## Data Availability

The data presented in this study are available upon request from the corresponding author. The data are not publicly available to protect the privacy of qualitative research participants.

## Supplementary Materials

The following supporting information can be downloaded at: https://www.mdpi.com/article/10.3390/healthcare13050465/s1, Figure S1: NEPSY-II auditory attention scores, Figure S2: NEPSY-II response set scores, Figure S3: Overall attention scores, Figure S4: Sustained attention scores, Figure S5: Selective attention scores, Figure S6: Alternating attention scores.

## References

[1] Cai R.Y., Richdale A.L., Uljarević M., Dissanayake C., Samson A.C. (2018). Emotion regulation in autism spectrum disorder: Where we are and where we need to go. Autism Res..

[2] Lord C., Brugha T.S., Charman T., Cusack J., Dumas G., Frazier T., Jones E.J.H., Pickles A., State M.W., Taylor J.L. (2020). Autism spectrum disorder. Nat. Rev. Dis. Primers.

[3] Janzen T.B., Thaut M.H. (2018). Rethinking the role of music in the neurodevelopment of autism spectrum disorder. Music. Sci..

[4] Deco G., Thiele A. (2009). Attention–oscillations and neuropharmacology. Eur. J. Neurosci..

[5] Bruinsma Y., Koegel R.L., Koegel L.K. (2004). Joint attention and children with autism: A review of the literature. Ment. Retard. Dev. Disabil. Res. Rev..

[6] Allen G., Courchesne E. (2001). Attention function and dysfunction in autism. Front. Biosci..

[7] Sohlberg M.M., Mateer C.A. (2001). Improving attention and managing attentional problems: Adapting rehabilitation techniques to adults with ADD. Ann. N. Y. Acad. Sci..

[8] Gross J.J. (2015). Emotion regulation: Current status and future prospects. Psychol. Inq..

[9] Koole S.L., Forgas J.P., Baumeister R.F., Tice D.M. (2009). Does emotion regulation help or hurt self-regulation?. Psychology of Self-Regulation: Cognitive, Affective, and Motivational Processes.

[10] Mazefsky C.A., Herrington J., Siegel M., Scarpa A., Maddox B.B., Scahill L., White S.W. (2013). The role of emotion regulation in autism spectrum disorder. J. Am. Acad. Child Adolesc. Psychiatry.

[11] Webb T.L., Miles E., Sheeran P. (2012). Dealing with feeling: A meta-analysis of the effectiveness of strategies derived from the process model of emotion regulation. Psychol. Bull..

[12] Porges S.W. (2017). The Pocket Guide to the Polyvagal Theory: The Transformative Power of Feeling Safe.

[13] Berger D. (2002). Music therapy in the realm of sensory integration. Music Therapy, Sensory Integration and the Autistic Child.

[14] Leitch L. (2017). Action steps using ACEs and trauma-informed care: A resilience model. Health Justice.

[15] Lutz A., Slagter H.A., Dunne J.D., Davidson R.J. (2008). Attention regulation and monitoring in meditation. Trends Cogn. Sci..

[16] Molnar-Szakacs I., Heaton P. (2012). Music: A unique window into the world of autism. Ann. N. Y. Acad. Sci..

[17] Koelsch S. (2014). Brain correlates of music-evoked emotions. Nat. Rev. Neurosci..

[18] Wigram T., Gold C. (2006). Music therapy in the assessment and treatment of autistic spectrum disorder: Clinical application and research evidence. Child Care Health Dev..

[19] Thaut M.H., Hoemberg V. (2014). Handbook of Neurologic Music Therapy.

[20] Koegel R.L., Koegel L.K., Carter C.M. (1999). Pivotal teaching interactions for children with autism. Sch. Psychol. Rev..

[21] Pellicano E., Burr D. (2012). When the world becomes ‘too real’: A Bayesian explanation of autistic perception. Trends Cogn. Sci..

[22] Robertson C.E., Baron-Cohen S. (2017). Sensory perception in autism. Nat. Rev. Neurosci..

[23] Bhatara A., Quintin E.M., Heaton P., Fombonne E., Levitin D.J. (2009). The effect of music on social attribution in adolescents with autism spectrum disorders. Child Neuropsychol..

[24] Patel A.D. (2008). Music, Language, and the Brain.

[25] Brown S., Martinez M.J., Parsons L.M. (2006). Music and language side by side in the brain: A PET study of the generation of melodies and sentences. Eur. J. Neurosci..

[26] Salimpoor V.N., Benovoy M., Larcher K., Dagher A., Zatorre R.J. (2011). Anatomically distinct dopamine release during anticipation and experience of peak emotion to music. Nat. Neurosci..

[27] Damasio A.R. (1994). Descartes’ Error: Emotion, Reason, and the Human Brain.

[28] Davidson R.J. (2003). Affective neuroscience and psychophysiology: Toward a synthesis. Psychophysiology.

[29] Greenfield S. (2000). The Private Life of the Brain.

[30] Overy K., Molnar-Szakacs I. (2009). Being together in time: Musical experience and the mirror neuron system. Music. Percept..

[31] Charcot J.M. (2011). Vibratory therapeutics: The application of rapid and continuous vibrations to the treatment of certain diseases of the nervous system. 1892. J. Nerv. Ment. Dis..

[32] Campbell E.A. (2019). Vibroacoustic Treatment and Self-Care for Managing the Chronic Pain Experience: An Operational Model. Ph.D. Thesis.

[33] Skille O. (1989). VibroAcoustic Therapy. Music. Ther..

[34] Buzsáki G., Draguhn A. (2004). Neuronal oscillations in cortical networks. Science.

[35] Bartel L.R., Chen R., Alain C., Ross B. (2017). Vibroacoustic stimulation and brain oscillation: From basic research to clinical application. Music. Med..

[36] Fauzan N., Amran N.H. (2015). Brain waves and connectivity of autism spectrum disorders. Procedia-Soc. Behav. Sci..

[37] Amini Shirazi N., Rezayi S., Asaseh M., Azizi M.P. (2023). Effectiveness of the Integrated Rehabilitation Program Based on Vibroacoustics and Virtual Reality on the Visual Processing of Children with Autism: Treatment Reports of Five Patients. J. Gorgan Univ. Med. Sci..

[38] Lundqvist L.O., Andersson G., Viding J. (2009). Effects of vibroacoustic music on challenging behaviors in individuals with autism and developmental disabilities. Res. Autism Spectr. Disord..

[39] Punkanen M., Nyberg M., Savela T. (2017). Vibroacoustic Therapy in the treatment of developmental trauma: Developing safety through vibrations. Music. Med..

[40] Ahonen H., Deek P., Kroeker J. (2012). Low frequency sound treatment promoting physical and emotional relaxation: A qualitative study. Int. J. Psychosoc. Rehabil..

[41] Chandrasekaran C., Ghazanfar A.A. (2009). Different neural frequency bands integrate faces and voices differently in the superior temporal sulcus. J. Neurophysiol..

[42] Creswell J.W., Clark V.L.P. (2018). Designing and Conducting Mixed Methods Research.

[43] Morgan D.L. (2007). Paradigms lost and pragmatism regained: Methodological implications of combining qualitative and quantitative methods. J. Mix. Methods Res..

[44] Ogbonnaya U.I., Awuah F.K. (2019). Quintile ranking of schools in South Africa and learners’ achievement in probability. Stat. Educ. Res. J..

[45] Moore J. (2024). Vibroacoustic Therapy and its Effects on the Attention of Children with Autism Spectrum Disorder. Master’s Thesis.

[46] Korkman M., Kirk U., Kemp S. (2007). NEPSY-Second Edition (NEPSY-II).

[47] Brooks B.L., Sherman E.M., Strauss E. (2009). NEPSY-II: A Developmental Neuropsychological Assessment. Child Neuropsychol..

[48] Davis J.L., Matthews R.N. (2010). NEPSY-II Review: Korkman, M.; Kirk, U.; Kemp, S. (2007). NEPSY—Second Edition (NEPSY-II). San Antonio, TX: Harcourt Assessment. J. Psychoeduc. Assess..

[49] Bean J.L., Eigsti I.M. (2012). Assessment of joint attention in school-age children and adolescents. Res. Autism Spectr. Disord..

[50] Dos Santos A., Wagner C. (2018). Musical elicitation methods: Insights from a study with becoming-adolescents referred to group music therapy for aggression. Int. J. Qual. Methods.

[51] Bradt J., Burns D.S., Creswell J.W. (2013). Mixed methods research in music therapy research. J. Music Ther..

[52] Bryman A. (2013). Quality issues in mixed methods research. Talk to The White Rose Social Science DTC Second Annual Spring Conference.

[53] Ziadat A.H. (2022). Effect of online-based physical activity vs. art activity on the joint attention of students with ASD. Int. J. Instr..

[54] Calamia M., Markon K., Tranel D. (2012). Scoring higher the second time around: Meta-analyses of practice effects in neuropsychological assessment. Clin. Neuropsychol..

[55] Borsboom D., Mellenbergh G.J., Van Heerden J. (2004). The concept of validity. Psychol. Rev..

[56] Heale R., Twycross A. (2015). Validity and reliability in quantitative studies. Evid. Based Nurs..

[57] Kim J., Wigram T., Gold C. (2008). The effects of improvisational music therapy on joint attention behaviors in autistic children: A randomized controlled study. J. Autism Dev. Disord..

[58] LaGasse A.B. (2014). Effects of a music therapy group intervention on enhancing social skills in children with autism. J. Music. Ther..

[59] Thompson G.A., McFerran K.S., Gold C. (2014). Family-centred music therapy to promote social engagement in young children with severe autism spectrum disorder: A randomized controlled study. Child Care Health Dev..

[60] Harder R. (2022). Joint attention and communication. Inquiry.

[61] Peiker I., David N., Schneider T.R., Nolte G., Schöttle D., Engel A.K. (2015). Perceptual integration deficits in autism spectrum disorders are associated with reduced interhemispheric gamma-band coherence. J. Neurosci..

[62] May P., Tiltinen H., Sinkkonen J., Näätänen R. (1994). Long-term stimulation attenuates the transient 40-Hz response. NeuroReport.

[63] Jensen O., Kaiser J., Lachaux J.P. (2007). Human gamma-frequency oscillations associated with attention and memory. Trends Neurosci..

[64] Kahlbrock N., Butz M., May E.S., Schnitzler A. (2012). Sustained gamma band synchronization in early visual areas reflects the level of selective attention. Neuroimage.

[65] Rüütel E., Ratnik M., Tamm E., Zilensk H. (2004). The experience of vibroacoustic therapy in the therapeutic intervention of adolescent girls. Nord. J. Music. Ther..

[66] Rodriguez M., Kross E. (2023). Sensory emotion regulation. Trends Cogn. Sci..

[67] Simhon V., Elefant C., Orkibi H. (2019). Associations between music and the sensory system: An integrative review for child therapy. Arts Psychother..

[68] Kilroy E., Aziz-Zadeh L., Cermak S. (2019). Ayres theories of autism and sensory integration revisited: What contemporary neuroscience has to say. Brain Sci..

[69] Dellapiazza F., Michelon C., Vernhet C., Muratori F., Blanc N., Picot M.C., Baghdadli A. (2021). Sensory processing related to attention in children with ASD, ADHD, or typical development: Results from the ELENA cohort. Eur. Child Adolesc. Psychiatry.

[70] Bridges H. (2015). Reframe Your Thinking Around Autism: How the Polyvagal Theory and Brain Plasticity Help Us Make Sense of Autism.

[71] Hartley M., Dorstyn D., Due C. (2019). Mindfulness for children and adults with autism spectrum disorder and their caregivers: A meta-analysis. J. Autism Dev. Disord..

[72] Kabat-Zinn J. (2003). Mindfulness-based stress reduction (MBSR). Constr. Hum. Sci..

[73] Richards K., Campenni C., Muse-Burke J. (2010). Self-care and well-being in mental health professionals: The mediating effects of self-awareness and mindfulness. J. Ment. Health Couns..

[74] Bieligmeyer S., Helmert E., Hautzinger M., Vagedes J. (2018). Feeling the sound—Short-term effect of a vibroacoustic music intervention on well-being and subjectively assessed warmth distribution in cancer patients—A randomized controlled trial. Complement. Ther. Med..

[75] Vilímek Z., Kantor J., Kořínková J. (2021). The impact of vibroacoustic therapy on subjective perception of university students—Mixed design pilot study. Univers. J. Educ. Res..

[76] Ellis P. (2004). Vibroacoustic Sound Therapy: Case Studies with Children with Profound and Multiple Learning Difficulties and the Elderly in Long-Term Residential Care. Medical and Care Compunetics.

[77] Mendes C.G., Diniz L.A., Marques Miranda D. (2021). Does music listening affect attention? A literature review. Dev. Neuropsychol..

[78] Mahraun D. (2004). The Influence of Music and Rhythm on a Sustained Attention Task in Children with Autism. Master’s Thesis.

[79] Clayton M.S., Yeung N., Kadosh R.C. (2015). The roles of cortical oscillations in sustained attention. Trends Cogn. Sci..

[80] Voloh B., Valiante T.A., Everling S., Womelsdorf T. (2015). Theta–gamma coordination between anterior cingulate and prefrontal cortex indexes correct attention shifts. Proc. Natl. Acad. Sci. USA.

[81] Button K.S., Ioannidis J.P., Mokrysz C., Nosek B.A., Flint J., Robinson E.S., Munafò M.R. (2013). Power failure: Why small sample size undermines the reliability of neuroscience. Nat. Rev. Neurosci..

